# Structural organization and functional divergence of high isoelectric point α-amylase genes in bread wheat (*Triticum aestivum* L.) and barley (*Hordeum vulgare* L.)

**DOI:** 10.1186/s12863-019-0732-1

**Published:** 2019-03-07

**Authors:** Liangliang Ju, Guangbing Deng, Junjun Liang, Haili Zhang, Qiao Li, Zhifen Pan, Maoqun Yu, Hai Long

**Affiliations:** 1 Chengdu Institute of Biology, Chinese Academy of Sciences, Chengdu, 610041 China; 2 University of Chinese Academy of Sciences, Beijing, 100049 China

**Keywords:** α-Amylase, Isoelectric point, Gene duplication, Chromosomal rearrangement, Functional divergence

## Abstract

**Background:**

High isoelectric point α-amylase genes (*Amy1*) play major roles during cereal seed germination, and are associated with unacceptable high residual α-amylase activities in ripe wheat grains. However, in wheat and barley, due to extremely high homology of duplicated copies, and large and complex genome background, the knowledge on this multigene family is limited.

**Results:**

In the present work, we identified a total of 41 *Amy1* genes among 13 investigated grasses. By using genomic resources and experimental validation, the exact copy numbers and chromosomal locations in wheat and barley were determined. Phylogenetic and syntenic analyses revealed tandem gene duplication and chromosomal rearrangement leading to separation of *Amy1* into two distinct loci, *Amy1θ* and *Amy1λ*. The divergence of *Amy1λ* from *Amy1θ* was driven by adaptive selection pressures performed on two amino acids, Arg_97_ and Asn_233_ (*P* > 0.95*). The predicted protein structural alteration caused by substitution of Asp_233_Asn in the conserved starch binding surface site, and significantly expressional differentiation during seed germination and grain development provided evidence of functional divergence between *Amy1θ* and *Amy1λ* genes. We screened out candidate copies (*TaAmy1-A1/A2* and *TaAmy1-D1*) associated with high residual α-amylase activities in ripe grains. Furthermore, we proposed an evolutionary model for expansion dynamics of *Amy1* genes.

**Conclusions:**

Our study provides comprehensive analyses of the *Amy1* multigene family, and defines the fixation of two spatially structural *Amy1* loci in wheat and barley. Potential functional divergence between them is reflected by their sequence features and expressional patterns, and driven by gene duplication, chromosome rearrangement and natural selections during gene family evolution. Furthermore, the discrimination of differentially effective copies during seed germination and/or grain development will provide guidance to manipulation of α-amylase activity in wheat and barley breeding for better yield and processing properties.

**Electronic supplementary material:**

The online version of this article (10.1186/s12863-019-0732-1) contains supplementary material, which is available to authorized users.

## Background

Alpha-amylase (α-D-1,4-glucan-4-glucanohydrolases, EC 3.2.1.1) is of critical importance to the breakdown of starch granules during seed germination [1–5]. It catalyzes the hydrolysis of internal α-D-1,4-glucosidic linkages in large polysaccharides to yield maltose and maltodextrin products [6]. In cereal crops, such as bread wheat and barley, two main isoforms of α-amylases have been extensively investigated during seed germination or in the gibberellic acid (GA_3_)-induced aleurone layers [1–5]. They were conserved in the biosynthesis and secretion from the scutellar epithelium and the aleurone layer to the starchy endosperm [7, 8]. Major differences in physicochemical and biochemical properties (i.e., sensitivity to Ca^2+^, stability at low pH and under heat treatment, and charge and serological characteristics) could be used to distinguish the two isoforms, which were eventually classified as high-pI and low-pI isoforms on the basis of isoelectric point (pI). The nomenclatures of high-pI and low-pI isoforms have not been consistent in the literature. In most of the literatures [9–12], researchers assigned high-pI and low-pI α-amylases as symbols of α-Amy-1 (Amy1) and α-Amy-2 (Amy2), respectively. Oppositely, some other literatures separately designated them as Amy2 and Amy1 [13–15]. Meanwhile, some authors [16, 17] named low-pI isoform as type A and high-pI as type B. In this work, we are prone to adopt high-pI α-amylase as Amy1 and low-pI as Amy2.

High-pI isoform (Amy1) was triggered by the commencement of seed germination and produced in higher concentration than that of low-pI α-amylase (Amy2), which was characterized by its synthesis both in kernel development and during seed germination [18]. Protein crystal structures of Amy1 and Amy2 were very similar, each consisting of three domains: a central conserved (β/α)_8_-barrel domain (domain A), an additional domain B nested between β_3_ and α_3_ of domain A, and a five-stranded C-terminal β-sheet domain (domain C) [14, 15, 19]. Substrate binding analysis revealed a starch granule binding surface site (SBS1) and a highly conserved active site in both of them among cereal grains, and when replaced by thio-maltotetraose as substrate analogues, a new Amy2-specific surface binding site at domain C had been discovered [15, 20]. Additionally, a novel wheat α-amylase (TaAMY3) was reported and considered as the most abundant isoform compared with the other known α-amylases throughout grain development [21, 22]. The newly described isoform HvAMY4 did not subject any predicted transit peptide and detected in various plant tissues [23]. Therefore, these four categories of α-amylases seem to accomplish starch degradations in different developmental stages or in various plant tissues, although their controlling and digesting mechanisms are still unclear. Accordingly, in cereal crops, genes encoding α-amylases have been divided into three or four subfamilies, i.e., *TaAMY1* to *TaAMY3* in bread wheat [12], *HvAMY1* to *HvAMY4* in barley [23] and *OsAMY1* to *OsAMY3* in rice [12]. Recently, *AMY4* genes have been identified and added to wheat and barley [24, 25].

Because of functional importance to the transition from dormancy to germinating, and its association with high residual α-amylase activities in ripe wheat grains, *Amy1* genes have been of interest to plant biologists for many years. This structural locus was located on the long arms of chromosomes 6A, 6B and 6D in bread wheat [10], and 6H in barley [26], respectively. It was thought to be complicated, compound and multigenetic, either consisting of tightly linked copies or involving some degree of gene duplications [4, 10]. As a self-pollination plant, bread wheat (AABBDD) is a product of at least two rounds of polyploidization, consisting three closely related diploid progenitors: *T. urartu* (A genome), *Aegilops speltoides*-related species (B genome) and *Ae. tauschii* (D genome) [27, 28]. The redundancy afforded by the hexaploid nature of bread wheat also increases difficulties to fully evaluate this structural locus. Based on simultaneous studies of polyacrylamide gel electrophoresis (PAGE), isoelectric focusing (IEF) and restriction fragment length polymorphism (RFLP), three *Amy1* copies, located on the chromosome 6R, were determined in the diploid genome of rye [29]. Khursheed and Rogers cloned two types of barley α-amylase genes (*Amy6–4* and *Amy46*) belonging to the high-pI multigene family, and confirmed their different mRNA levels in the GA_3_-induced aleurone cells [30]. In addition, functional analysis of the *Amy6–4* promoter region indicated that the gibberellic acid response elements (GARE) was comprised of three conserved *cis*-acting boxes (‘pyrimidine’ box, ‘taacaaac’ box and ‘tatccac/t’ box) [31, 32]. Through binding to the central element (‘taacaaac’ box), the GA_3_-regulated transcription factor (GAMyb) activated transcription of *Amy1* genes [33]. In rice, the *RAmy1* subfamily (*RAmy1A*, *RAmy1B* and *RAmy1C*) consists of genes corresponding to the *Amy1* classes of barley and wheat [12]. *RAmy1A* (*AmyI-1*) transcript was most abundant in germinating seeds and involved in the degradation of plastid starch granules [34, 35]. Recently, as reviewed by Mares and Mrva [36], pre-harvest sprouting (PHS) and late maturity α-amylase (LMA) were characterized by high levels of α-amylases in ripe wheat grains. Wheat lines with abnormal accumulation of α-amylases lowered whole meal falling numbers and resulted in reduced starch viscosity and poor flour quality [36–38]. Yang et al. [39] and Cheng et al. [40] isolated *Amy1* genes involved in PHS-affected and LMA-affected lines, and detected high expression of *Amy1* genes was strongly correlated with high levels of high-pI α-amylases.

Despite considerable progresses have been made in understanding the characteristics of *Amy1* genes, to date, comprehensive analysis focusing on this multigene family still lacks. It has been well established that three major grass subfamilies (Pooideae, Ehrhartoideae and Panicoideae) evolve from a common ancestral cereal genome with a basic number of five chromosomes [41, 42]. Together with recently released genome datasets of hexaploid wheat and barley, this inner circle model facilitates access to investigate the conserved block carrying this structural locus with multiple high-homology gene members, and help us to better explore their structural organization and expansion dynamics. Therefore, elucidating functional diversification of duplicated copies of this multigene family is needed for further practices of agronomic traits improvement and molecular design breeding.

In this paper, we firstly estimate the exact copy number of *Amy1* genes in grass. Then, we reconstruct the phylogeny and investigate structural organization. For further exploring evolutionary forces and understanding functional implications, we conduct potential natural selection tests, build three-dimensional (3D) protein homology structures, and quantify the expression profiles in depth from copy-specific levels.

## Methods

### Plant materials and tissue sampling

Chinese Spring (CS) (*T. aestivum*) and six nullisomic-tetrasomic (NT) lines for the homoeologous group 6, PI428191 (*T. urartu*), PI542268 (*Ae. speltoides*) and AS2404 (*Ae. tauschii*) were used for DNA extraction and cloning experiments; seeds were incubated at room temperature for 5 to 7 days under darkness condition. Based on preliminary phenotyping under three different field conditions (Sichuan shuangliu in 2014/2015, Sichuan shifang and Yunnan yuanmou in 2014/2015), three wheat cultivars (Guinong19, Mianmai43 and Jinan17) with high residual α-amylase activities in ripe grains and the absence of sprouting, and three landraces (Honghuamai, Siqiangxiaomai and Guangguangtou) with extreme low activities were selected for measurement of total α-amylase activities and preparation of mRNA in the developing grains. Spikes were tagged at anthesis and sampled at 10, 12, 14, 16, 18, 20, 22, 24, 26, 28, 30, 32, 34 and 36 days post anthesis (DPA) in Sichuan shuangliu from late-March to early-May in 2016. For germination sampling, seeds of bread wheat (cv. CS) and barley (cv. Morex) were incubated on a moist filter paper in petri dishes under darkness condition (25 °C); similar seedlings were collected at 12, 24, 36, 48 h (h) after seed imbibition. During growth, field management followed normal field operations. The plant materials used in the study are held in our own lab.

### Identification of *Amy1* genes in grass

Two approaches (molecular cloning and in silico analysis) were used to identify *Amy1* genes. Firstly, the public available *Amy1* sequences were collected from databases of bread wheat and other Triticeae species (https://urgi.versailles.inra.fr/blast/). To detect the *TaAmy1* copy number, primer pair *TaAmy1*-F/R was developed for cloning the full-length genomic sequences. Total genomic DNA was extracted following the cetyl trimethylammonium bromide (CTAB) method with minor modifications. PCR products were purified and sequenced using the same forward and reverse primers at Sangon Biotech (Shanghai, China). Due to limited Taq-polymerase fidelity, clones with sub-optimal quality were discarded for further analysis.

Two barley *Amy1* genes, *Amy6–4* (GenBank accession no. K02637) and *Amy46* (GenBank accession no. J04202) [30], were employed as query sequences to blast against the genomes of bread wheat (IWGSC WGA v0.4, https://urgi.versailles.inra.fr/blast_iwgsc/), barley (http://webblast.ipk-gatersleben.de/), rice (http://rice.plantbiology.msu.edu/) and other grasses (https://phytozome.jgi.doe.gov/) with default setting parameters. BLAST hits with an expectancy value (E-value) of zero were subjected to the second round of BLAST searches within the genomes from which they were identified. All the retrieved and cloned *Amy1* gene sequences were aligned with Clustal X 2.0 [43] and manually modified with BioEdit v7.2 [44]. Exons and introns were positioned by aligning full genomic sequences and their corresponding coding sequences, and visualized by the GSDS 2.0 server [45]. The pI values of putative amino acids were calculated using online computation tool (http://web.expasy.org/compute_pi/). Subcellular localization and cleavage site prediction were performed using the CBS TargetP software [46, 47].

### Phylogenetic reconstruction and positive selection detection

Full-length coding sequences were used for phylogenetic analysis. Maximum likelihood trees were constructed by MEGA7 software [48] using the Tamura 3-parameter + GAMMA substitution model [49], the best fitting model as determined by the “Find Best DNA/Protein Models” function in MEGA7. All positions containing gaps and missing data were eliminated. Branch supports attached to each node were inferred from 1000 bootstrap replicates and values less than 75% were collapsed. Putative amino acids were used for estimating the genetic distance of *Amy1* genes as described by Jones et al. [50]. Synonymous (Ks) and non-synonymous (Ka) substitution rates of paralogs and orthologs were calculated as described previously [51]. Codon-based substitution models, M0 (one-ratio) and Two-ratios [52, 53], M1a (Neutral) and M2a (Selection) [54, 55], M3 (discrete), M7 (beta) and M8 (beta & omega) [56], Model A and Model B [57, 58], were applied to detect branches or sites under positive selection, which were conducted using the codeml program [52] implemented in the PAML package [59]. Divergence time (T) was obtained using a synonymous rate of 6.5 × 10^− 9^ substitutions per site per year [60–62] as T = Ks / (2 × 6.5 × 10^− 9^).

### Synteny investigation and repeats annotation

Chromosomes anchoring *Amy1* loci were downloaded from online resources to construct a local genomic database. A total of 112 structural genes flanking the rice *Amy1* locus (LOC_Os02g52700 and LOC_Os02g52710) were used as query markers to search against the local database using the basic tool NCBI-BLAST-2.4.0+ [63]. Reciprocal blastp [64] was carried out to confirm the orthologous relationships between pairs of corresponding structural markers. Genomic segments covering these markers were selected for detecting gene orders and synteny relationships. The identification of repetitive elements was analyzed by using a local BLASTN search against the non-redundant dataset of Triticeae Repeats (http://botserv2.uzh.ch/kelldata/trep-db/index.html).

### Protein structure homology modeling

Two protein homologues, barley 1AMY [14] and rice 3WN6 [19], were extracted from the Protein Data Bank (PDB) archive (http://www.rcsb.org/pdb/home/home.do). Primary sequence alignment (not including the signal peptides) and secondary structure analysis were performed and displayed using the ESPript 3.0 server [65]. Annotation details of amino acids were inferred from the National Center for Biotechnology Information (NCBI) conserved domain database [66]. Protein structure homology modeling was built in the workplace of SWISS-MODEL [67–69] based on the molecular structure of a barley α-amylase-inhibitor complex (PDB ID: 1BG9) [70]. UCSF Chimera [71] was used for visualization and analysis of the resulting models.

### Alpha-amylase assay

Developing grains were removed from the central part of the spikes. The wholemeal samples were dehydrated using VirTis freeze drying equipment and Lyophilizers (SP SCIENTIFIC). Alpha-amylase activity was determined following protocols of Whan et al. [22]. Data was expressed in ceralpha unit (CU) per g four or μg of protein as determined by Bradford assays [72] on the CERALPHA extracts.

### RNA extraction and qRT-PCR

Total RNA from germinating seeds and developing grains was extracted using a TaKaRa RNAiso Plus kit (http://www.takara.com.cn/). RNA was quantified using NANODROP 2000c. Approximately 2 μg of total RNA was used for reverse transcription and cDNA synthesis using TaKaRa Reverse Transcriptase M-MLV (RNase H-) following the supplier recommendations. Real time PCR was performed using SYBR green PCR master mix (Bio-Rad) in a 20-μL reaction system on CFX Connect™ Real-Time PCR Detection System (Bio-Rad). RT-PCR data were extracted using CFX Manager 3.1 software (Bio-Rad) and analyzed by 2^−ΔCT^ method. The expression of *TaActin* [22, 73] was used as an internal control for normalization. All the primers used in this work were listed in Additional file 1.

## Results

### Identification of *Amy1* genes in grass

As summarized in Table 1, a total of 41 *Amy1* genes were identified from the 13 investigated grass species. In bread wheat, there are three full-length copies in each of A and D genomes, and six copies (five full-length and one truncated *TaAmy1-B6*) in B genome. We also isolated these A- and D-genome copies in *T. aestivum*, *T. urartu* and *Ae. tauschii*, respectively. Multiple sequence alignment indicated that the genomic sequences of *TaAmy1-D1*, *TaAmy1-D2* and *TaAmy1-D3* were identical to *AetAmy1-D1*, *AetAmy1-D2* and *AetAmy1-D3*, respectively. Pairwise identities were 98.7% between *TaAmy1-A1* and *TuAmy1-A1*, 99.7% between *TaAmy1-A2* and *TuAmy1-A2*, and 99.1% between *TaAmy1-A3* and *TuAmy1-A3*. As for the B genome, we cloned three full-length copies (*TaAmy1-B1*, *TaAmy1-B2* and *TaAmy1-B3*) in *T. aestivum* and six (*AesAmy1-B1* to *AesAmy1-B6*) in *Ae. speltoides*, and pairwise identities (88.1 to 97.8%) were significantly lower than that of A and D genomes. All the isolated *Amy1* sequences are clustered in Additional file 2. The existence of *TaAmy1-B6* was verified by using primer pair *TaAmy1-B6*-F/R (Additional file 3A).

**Table 1:** Summary information of *Amy1* genes identified in grass species

Species	Gene name	gDNA (bp)	Chr^d^	Protein (aa)	pI	TargetP prediction	
						SP^e^	TPlen^f^
*Triticum aestivum* (CS)^a^	*TaAmy1-A1* ^b^	1472	6A	427	5.83	0.994	24
	*TaAmy1-A2* ^b^	1470	6A	427	6.00	0.995	24
	*TaAmy1-A3*	1773	6A	427	5.84	0.993	24
	*TaAmy1-B1* ^b^	1475	6B	427	5.92	0.990	24
	*TaAmy1-B2* ^b^	1468	6B	427	6.01	0.992	24
	*TaAmy1-B3* ^b^	1491	6B	425	5.84	0.989	22
	*TaAmy1-B4*	1494	6B	425	5.84	0.989	22
	*TaAmy1-B5*	1494	6B	425	5.84	0.989	22
	*TaAmy1-B6* ^c^	752	6B	–	–	–	–
	*TaAmy1-D1* ^b^	1478	6D	427	5.92	0.993	24
	*TaAmy1-D2* ^b^	1476	6D	425	6.10	0.990	24
	*TaAmy1-D3* ^b^	1757	6D	427	5.92	0.993	24
*Triticum urartu* (PI428191)^a^	*TuAmy1-A1*	1479	6A	427	5.83	0.995	24
	*TuAmy1-A2*	1473	6A	427	6.00	0.995	24
	*TuAmy1-A3*	1774	6A	427	5.84	0.993	24
*Aegilops speltoides* (PI542268)^a^	*AesAmy1-B1*	1475	6B	425	5.88	0.990	22
	*AesAmy1-B2*	1471	6B	425	5.92	0.990	22
	*AesAmy1-B3*	1478	6B	427	5.88	0.992	24
	*AesAmy1-B4*	1490	6B	427	5.81	0.993	24
	*AesAmy1-B5*	1510	6B	425	5.81	0.989	22
	*AesAmy1-B6*	1752	6B	427	5.92	0.993	24
*Aegilops tauschii* (AS2404)^a^	*AetAmy1-D1*	1478	6D	427	5.92	0.993	24
	*AetAmy1-D2*	1476	6D	425	6.10	0.990	24
	*AetAmy1-D3*	1757	6D	427	5.92	0.993	24
*Hordeum vulgare*	*HvAmy1–1*	1471	6H	427	5.79	0.993	24
	*HvAmy1–2*	1471	6H	427	5.79	0.993	24
	*HvAmy1–3*	1471	6H	427	5.79	0.993	24
	*HvAmy1–4*	1485	6H	427	5.74	0.994	24
	*HvAmy1–5*	1471	–	427	5.79	0.993	24
	*HvAmy1–6* ^c^	682	6H	–	–	–	–
*Brachypodium distachyon*	*BdAmy1*	1726	3	428	5.60	0.989	24
*Brachypodium stacei*	*BsAmy1*	1650	4	428	5.46	0.991	24
*Oryza sativa*	*OsAmy1–1*	1567	2	428	5.07	0.994	25
	*OsAmy1–2*	1573	2	428	5.06	0.994	25
*Panicum hallii*	*PhAmy1–1*	1598	1	427	5.12	0.987	24
	*PhAmy1–2*	1590	1	427	4.98	0.986	23
*Setaria italica*	*SiAmy1*	1463	1	427	5.08	0.991	24
*Setaria viridis*	*SvAmy1*	1463	1	427	5.08	0.991	24
*Sorghum bicolor*	*SbAmy1–1*	1563	4	428	4.99	0.993	24
	*SbAmy1–2*	1860	4	428	6.16	0.995	24
*Zea mays*	*ZmAmy1*	1651	5	428	5.18	0.986	24

^a^Plant materials used for *Amy1* isolation. ^b^Eight cloned full-length *Amy1* copies in CS. ^c^The truncated copies from wheat and barley. ^d^Chromosomes. ^e^Secretory pathway. ^f^The length of signal peptide

In barley, we identified five copies located on the chromosome 6H, and three copies with unknown chromosomal locations. Because of existence of gapped sequences in the coding region, two of them were discarded for further analyses. Similar to *TaAmy1-B6*, *HvAmy1–6* was also a truncated copy with approximate 682 bp gene sequences retained. Additionally, in silico analysis indicated that two copies were found in each of *O. sativa*, *P. hallii* and *S. bicolor*. Only a single copy was identified in each of *B. distachyon*, *B. stacei*, *S. italica*, *S. viridis* and *Z. mays*.

**Fig. 1:**
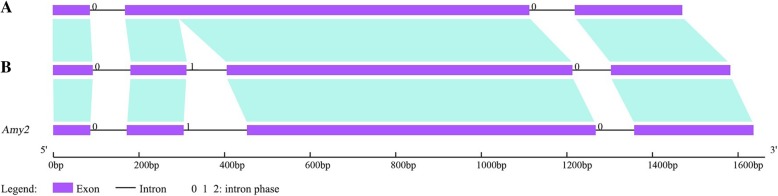
Schematic representation of two types of exon-intron structures. Pattern A was present in species of *T. aestivum*, *T. urartu*, *Ae. speltoides*, *Ae. tauschii*, *H. vulgare*, *S. italica* and *S. viridis*. Pattern B was found in *B. distachyon*, *B. stacei*, *O. sativa*, *P. hallii*, *S. bicolor* and *Z. mays*. Lengths of exons and introns were displayed proportionally. Because *Amy1* genes shared high sequence identities with *Amy2*, we used the *Amy2* exon-intron structure as a reference

Exon and intron analysis revealed two types of exon-intron structures (Fig. 1). Pattern A contained three exons and two introns, whereas the second exon of pattern B was interrupted by a middle intron and separated into two exons. Furthermore, we calculated theoretical pI values, which range from 4.98 to 6.10. All the full-length *Amy1* genes showed strong signals in secretory pathway (SP ≥ 0.986), and the length of signal peptides varied from 22 to 25 amino acids.

### Phylogenetic and syntenic relationships

**Fig. 2:**
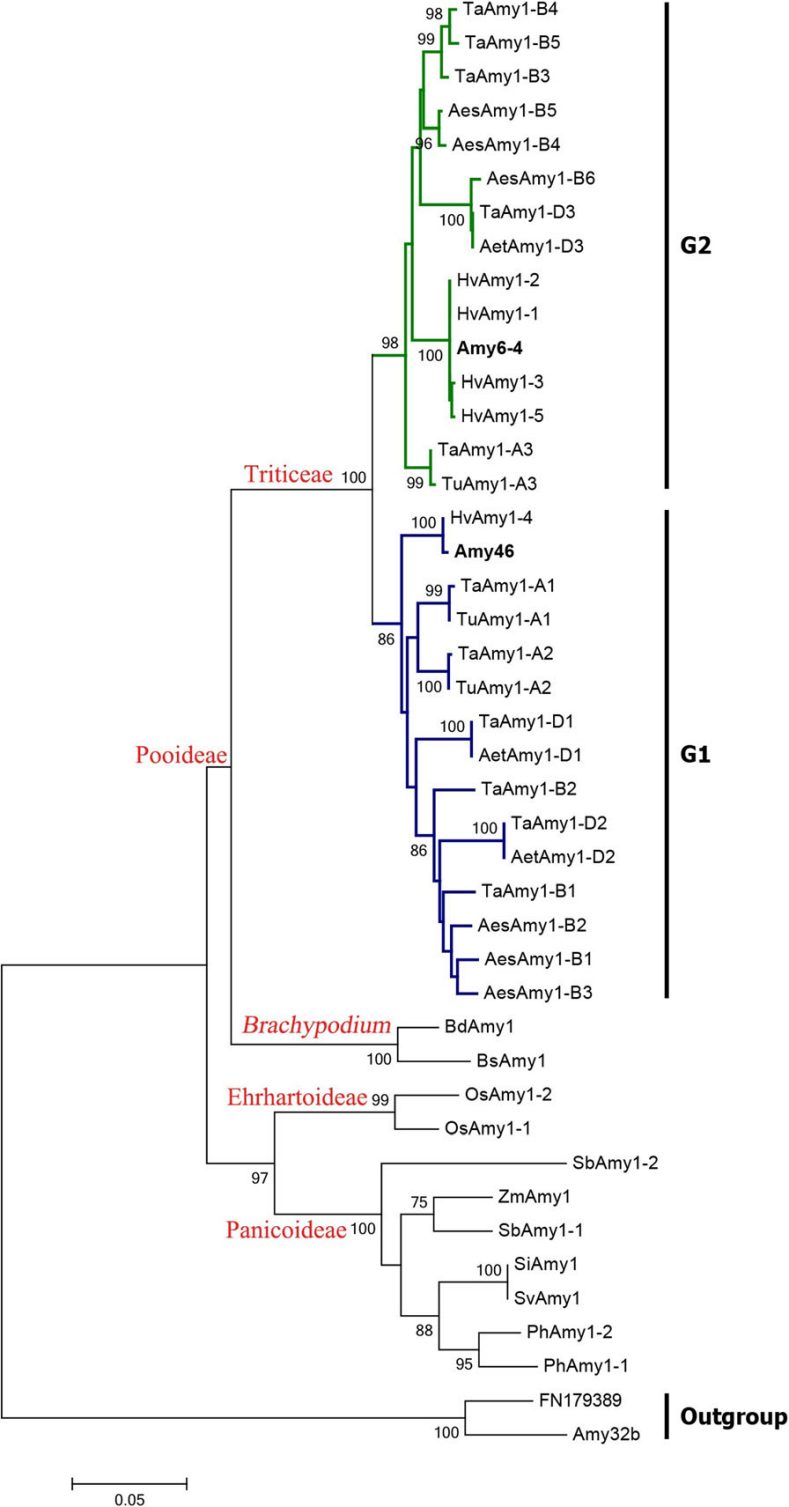
Phylogenetic analysis of grass *Amy1* genes. Forty-three protein-coding sequences from 13 species were involved. The tree is rooted on midpoint and drawn to scale, with branch lengths measured in the number of substitutions per site. A discrete Gamma distribution was used to model evolutionary rate differences among sites [5 categories (+G, parameter = 0.4987)]. Two low-pI *Amy2* genes, FN179389 [23] and *Amy32b* (GenBank accession no. X05166) were used as outgroup

To determine evolutionary relationship of *Amy1* family in grass, the phylogeny was reconstructed based on the maximum-likelihood method. The phylogenetic tree segregated *Amy1* genes into three major clades with high bootstrap supports (99% or 100%), corresponding to the three subfamilies (Pooideae, Ehrhartoideae and Panicoideae) in grass (Fig. 2). In the Pooideae-Triticeae clade, 28 identified sequences from five Triticeae species were clustered into two groups: Group 1 (G1) and Group 2 (G2). G1 contained 14 members: two in each of *T. urartu*, *Ae. tauschii*, genomes A, B and D of wheat, three in *Ae. speltoides*, and one in *H. vulgare*. G2 contained one in each of *T. urartu*, *Ae. tauschii*, genomes A and D of wheat, three in each of *Ae. speltoides* and B genome, and four in *H. vulgare*. We further estimated the evolutionary distances. All the three clades (Pooideae-*Brachypodium*, Ehrhartoideae and Panicoideae) exhibited larger genetic distances with G1 than those with G2 (Additional file 4), suggesting that G2 might be the ancient archetype *Amy1* genes in Triticeae.

**Fig. 3:**
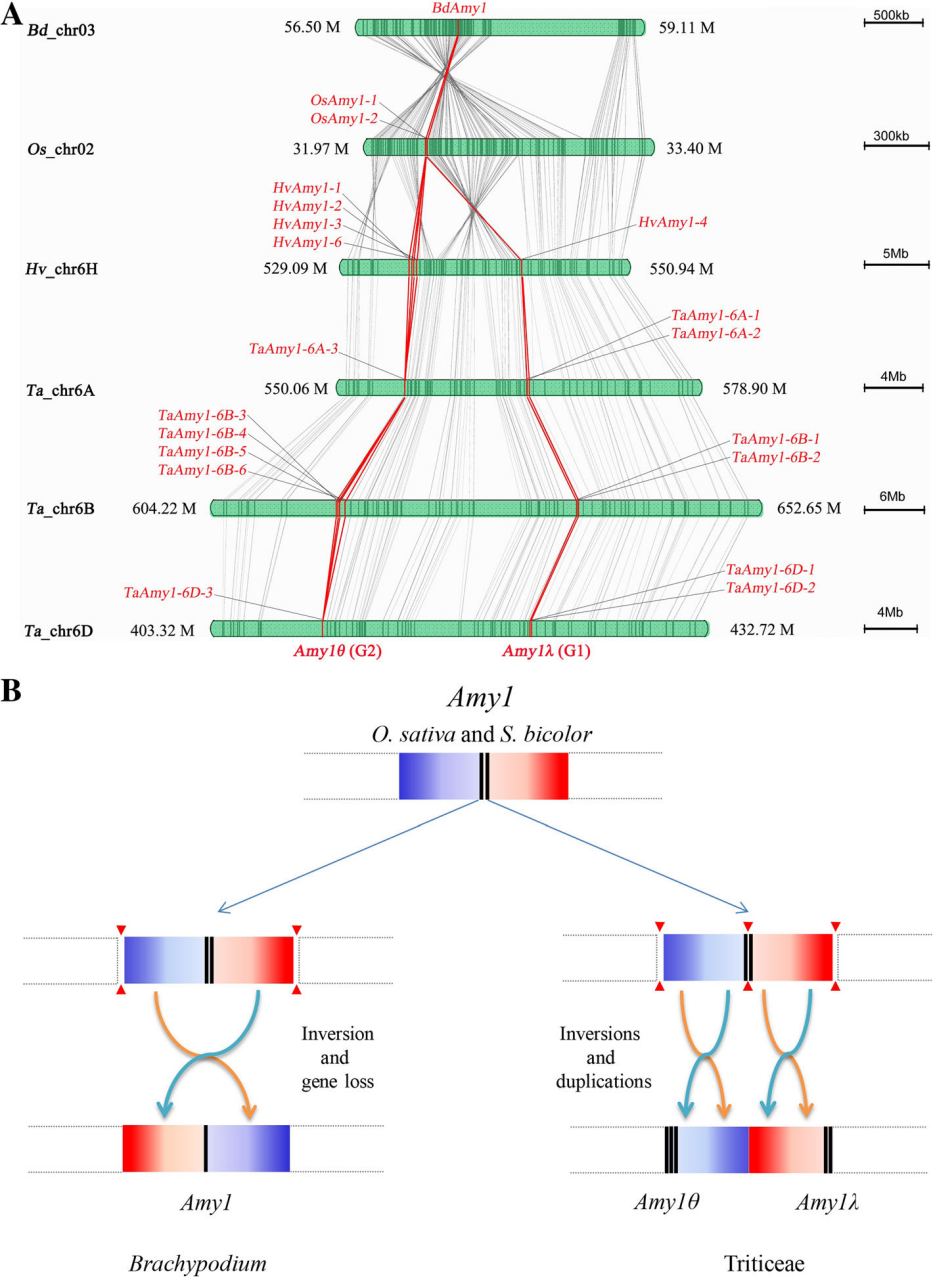
(**a**) Synteny relationships of genomic segments covering *Amy1* loci in rice, wheat, barley and *B. distachyon*. The *Amy1* genes were red highlighted and scale bars were proportional to the length of chromosomal segments. (**b**) Schematic illustration of structural variations of *Amy1* region

To make insights into structural organization of *Amy1* loci, the linear gene orders were analyzed across six grass genomes (Fig. 3A). The *Amy1* regions in wheat and barley shared highly conserved gene orders. However, compared to *Amy1* genes of rice located in the middle of segments, those of wheat and barley were dispersed on both ends of corresponding segments. The segments seemed to be separated into two parts, each of them was involved in an inversion event. A tandem gene duplication event was also found, which resulted in expansion of *Amy1* family (Fig. 3A, B). The two *Amy1* gene clusters apart from each other were corresponding to the phylogenetic groups G1 and G2, temporarily designated as *Amy1λ* and *Amy1θ*, respectively. Intervals between them, ranging from approximately 8.36 Mb on chromosome 6H to 21.42 Mb on chromosome 6B, were rich in repetitive elements such as long terminal repeat (LTR) retrotransposons and DNA transposons (Fig. 3A, Additional file 5).

### Divergence time of *Amy1λ* from *Amy1θ*

As mentioned above, a tandem gene duplication event, followed by chromosomal rearrangements, led to the fixation of *Amy1θ* and *Amy1λ* in genomes of wheat and barley. Considering a single *Amy1* copy in genera *Brachypodium*, this structural variation might occur prior to the divergence of wheat from barley (11.6 MYA) and after the divergence of wheat from *Brachypodium* (32–39 MYA) [42, 74]. To verify it, we calculated the pairwise mean synonymous substitution rates (dS) and estimated when this duplication event occurred. The divergence time of *Amy1λ* from *Amy1θ* occurred at approximately 36.3 ± 6.2 MYA in A genome (31.6 ± 5.7 MYA in *T. urartu*), 31.2 ± 5.6 MYA in B genome (39.4 ± 6.9 MYA in *Ae. speltoides*), 42.9 ± 7.7 MYA in D genome (42.9 ± 7.7 MYA in *Ae. tauschii*), and 36.0 ± 6.6 MYA in barley (Table 2). These results agreed with our hypothesis, and our estimates also suggested a higher substitution rate of *Amy1* genes than the average 6.5 × 10^− 9^ in grass nuclear genes.

**Table 2:** Estimation of duplication and divergence time between groups

Pairwise comparison	dS^a^	Average dS^a^	T (MYA^b^)
*TaAmy1-A3* vs *TaAmy1-A1*	0.5080 ± 0.0869	0.4725 ± 0.08095	36.3 ± 6.2
*TaAmy1-A3* vs *TaAmy1-A2*	0.4370 ± 0.0750		
*TuAmy1-A3* vs *TuAmy1-A1*	0.4143 ± 0.0745	0.4113 ± 0.07355	31.6 ± 5.7
*TuAmy1-A3* vs *TuAmy1-A2*	0.4082 ± 0.0726		
*TaAmy1-B4* vs *TaAmy1-B1*	0.3397 ± 0.0620	0.4062 ± 0.07242	31.2 ± 5.6
*TaAmy1-B4* vs *TaAmy1-B2*	0.3996 ± 0.0699		
*TaAmy1-B5* vs *TaAmy1-B1*	0.3568 ± 0.0641		
*TaAmy1-B5* vs *TaAmy1-B2*	0.4244 ± 0.0730		
*TaAmy1-B3* vs *TaAmy1-B1*	0.4334 ± 0.0805		
*TaAmy1-B3* vs *TaAmy1-B2*	0.4822 ± 0.0850		
*AesAmy1-B4* vs *AesAmy1-B1*	0.5588 ± 0.0946	0.5129 ± 0.09034	39.4 ± 6.9
*AesAmy1-B4* vs *AesAmy1-B2*	0.4935 ± 0.0871		
*AesAmy1-B4* vs *AesAmy1-B3*	0.4638 ± 0.0834		
*AesAmy1-B5* vs *AesAmy1-B1*	0.5355 ± 0.0901		
*AesAmy1-B5* vs *AesAmy1-B2*	0.4568 ± 0.0801		
*AesAmy1-B5* vs *AesAmy1-B3*	0.4763 ± 0.0863		
*AesAmy1-B6* vs *AesAmy1-B1*	0.6168 ± 0.1090		
*AesAmy1-B6* vs *AesAmy1-B2*	0.4839 ± 0.0836		
*AesAmy1-B6* vs *AesAmy1-B3*	0.5311 ± 0.0989		
*TaAmy1-D3* vs *TaAmy1-D1*	0.6974 ± 0.1287	0.5584 ± 0.1003	42.9 ± 7.7
*TaAmy1-D3* vs *TaAmy1-D2*	0.4193 ± 0.0719		
*AetAmy1-D3* vs *AetAmy1-D1*	0.6974 ± 0.1287	0.5584 ± 0.1000	42.9 ± 7.7
*AetAmy1-D3* vs *AetAmy1-D2*	0.4193 ± 0.0719		
*HvAmy1–4* vs *HvAmy1–1*	0.4682 ± 0.0858	0.4684 ± 0.0858	36.0 ± 6.6
*HvAmy1–4* vs *HvAmy1–2*	0.4682 ± 0.0858		
*HvAmy1–4* vs *HvAmy1–3*	0.4687 ± 0.0859		
*HvAmy1–4* vs *HvAmy1–5*	0.4687 ± 0.0859		

^a^Number of substitutions per synonymous site. ^b^Million years ago

### Potential natural selection on *Amy1λ* genes

We applied nine different codon-substitution models to detect selection pressures at individual sites along specific lineages. Results obtained were presented in Table 3. The one-ratio model (M0) produced an estimated ω_0_ = 0.0519. The two-ratio model assigned two different ω ratios for the foreground branch G1 (ω_1_ = 0.182) and for all other background branches (ω_0_ = 0.0513). Site-specific models indicated variable selective pressures among these 424 codons. For example, the M3 model (K = 2) fit the data better than the one-ratio model, the Likelihood Ratio Test (LRT) statistics were 2∆*ℓ* = 420, with *P* < 0.001 and df = 2. Both the branch models and site-specific models failed to detect sites under positive selection, and most sites appeared to be under strong purifying selection. The branch-site model A fit the data significantly better than M1a, the test statistics were 2∆*ℓ* = 3.6, with *P* = 0.06 and df = 1. Model B did not fit the data significantly better than M3 (discrete with K = 2) (2∆*ℓ* = 3.68, *P* = 0.16, df = 2), but it suggested a proportion of sites (19.7%) were under positive selection along the G1 branch with ω_2_ = 1.197. Both in Model A and Model B, four sites (56 V, 119R, 189 K, 254 N) have been detected under selection at a less significant level (0.50 < *P* < 0.95*) in the Bayes Empirical Bayes analysis (BEB), and sites 119R (His97Arg) and 254 N (Asp233Asn) were up to the significant level (*P* > 0.95*) in the Naive Empirical Bayes analysis (NEB).

**Table 3:** Analysis of potential natural selection analysis among *Amy1* genes

Model	*p* ^a^	*ℓ* ^b^	Estimates of Parameters	Positive Selected Sites
M0: one-ratio	1	− 7710.25	ω_0_ = 0.0519	NA^**c**^
Branch model				
Two-ratios	2	− 7708.92	ω_0_ = 0.0513, ω_1_ = 0.182	None
Site-specific model				
M1a: neutral	2	− 7606.65	*p*_*0*_ = 0.938 (*p*_*1*_ = 0.0620)	NA^**c**^
			ω_0_ = 0.0409 (ω_1_ = 1.000)	
M2a:selection	4	−7606.65	*p*_*0*_ = 0.938, *p*_*1*_ = 0.0620 (*p*_*2*_ = 0.000)	397S (*P* = 0.57)
			ω_0_ = 0.0409 (ω_1_ = 1.000), ω_2_ = 1.000	
M3: discrete (K = 2)	3	− 7500.24	*p*_*0*_ = 0.776 (*p*_*1*_ = 0.224)	None
			ω_0_ = 0.0131, ω_1_ = 0.219	
M3: discrete (K = 3)	5	− 7487.11	*p*_*0*_ = 0.705, *p*_*1*_ = 0.240 (*p*_*2*_ = 0.0549)	None
			ω_0_ = 0.009, ω_1_ = 0.131, ω_2_ = 0.446	
M7: beta	2	− 7490.53	*p* = 0.254 *q* = 3.432	NA^**c**^
M8: beta&ω > 1	4	− 7488.21	*p*_*0*_ = 0.989 (*p*_*1*_ = 0.0108)	None
			*p* = 0.281, *q* = 4.425, ω_s_ = 1.000	
Branch-site model				
Model A	3	− 7604.85	*p*_*0*_ = 0.878, *p*_*1*_ = 0.0484,	Foreground lineage ω_2_ (BEB^**d**^):
			(*p*_*2a*_ + *p*_*2b*_ = 0.0736)	56 V 119H 189 K 254D
			ω_2_ = 1.000	(0.70 < *P* < 0.95)
Model B	5	− 7498.40	*p*_*0*_ = 0.620,	Foreground lineage ω_2_ (NEB^**d**^):
			*p*_*1*_ = 0.183 (*p*_*2a*_ + *p*_*2b*_ = 0.197)	119H 254D (*P* > 0.95*)
			ω_0_ = 0.0125, ω_1_ = 0.216,	Foreground lineage ω_2_ (BEB^**d**^):
			ω_2_ = 1.197	56 V 119H 189 K 254D
				(0.50 < *P* < 0.85)

^a^The number of free parameters for ω distribution. ^b^Value of log likelihoods. ^c^Not allowed. ^d^*NEB* Naive Empirical Bayes, *BEB* Bayes Empirical Bayes. Amino acids refer to the first sequence: BdAmy1

### Homology modeling

**Fig. 4:**
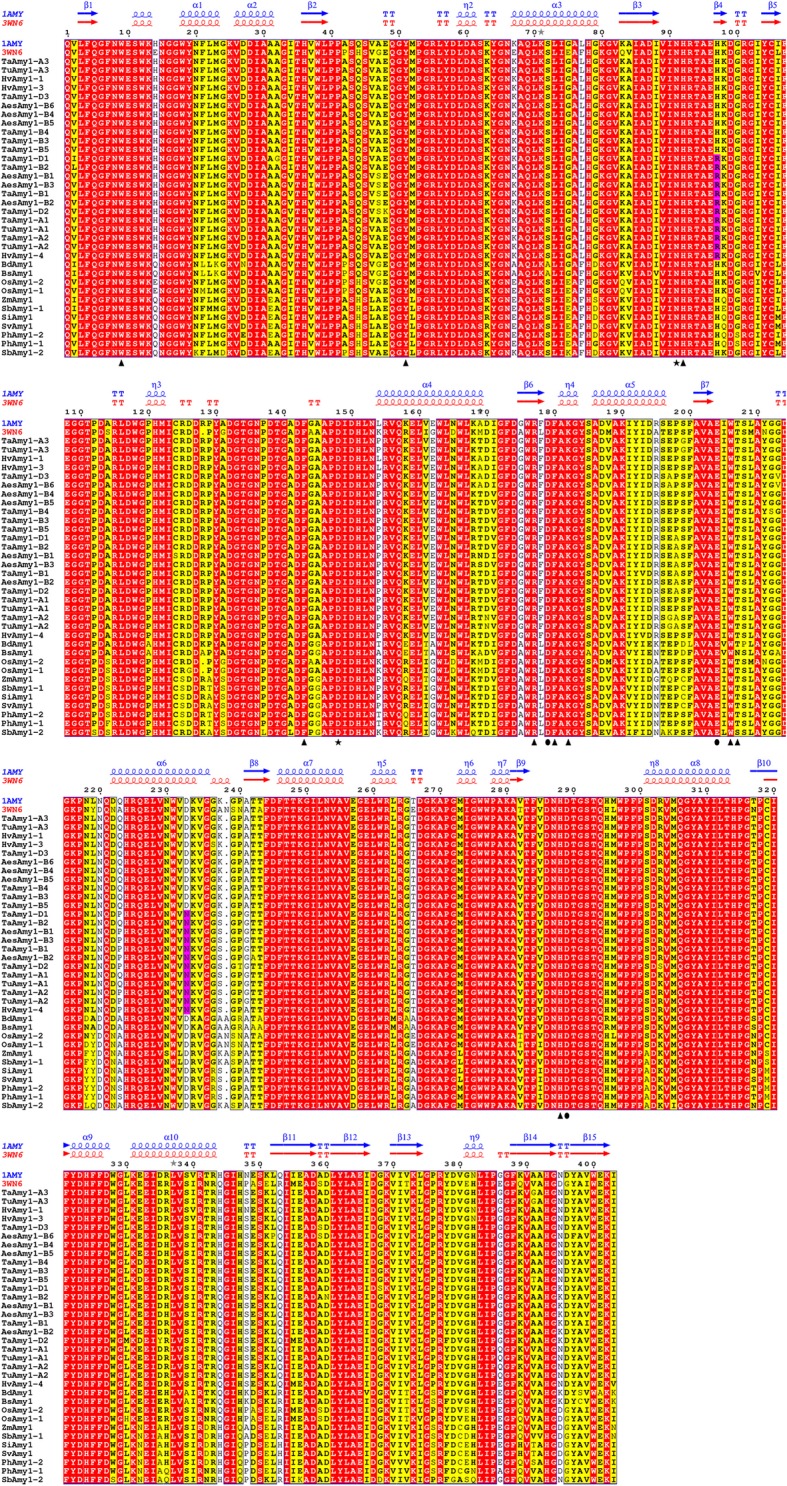
Protein sequence alignment of 403 amino acid residues. Secondary structure prediction was based on structures of barley 1AMY (blue) and rice 3WN6 (red) with α-helices displayed as coils, β-strands as arrows, strict β-turns as TT letters, active sites as triangles, Ca^2+^ binding sites as stars and catalytic sites as circles. Domain A: a (β/α)_8_-barrel of 286 residues, domain B: 64 residues, connecting strand β_3_ and helix α_4_ of the barrel, domain C: 53 residues forming a five stranded anti-parallel β-sheet. Two amino acids (Arg_97_ and Asn_233_), specific to Amy1λ proteins, were highlighted in pink

To check if there exists some functional divergence between proteins of Amy1θ and Amy1λ, initially, we analyzed the primary and secondary structures. We found two group-specific amino acid substitutions, His97Arg and Asp233Asn, located on the 4th β-strand in domain B and the 6th α-helix in domain A, respectively (Fig. 4). Then we built protein models, and found that when Asp_233_ was replaced by Asn_233_, Amy1λ proteins failed to form the α-amylase-acarbose inhibitor complex (Amy1-AF1) in the SBS1 region (Additional file 6).

### Expression profiles of *Amy1* genes

**Fig. 5:**
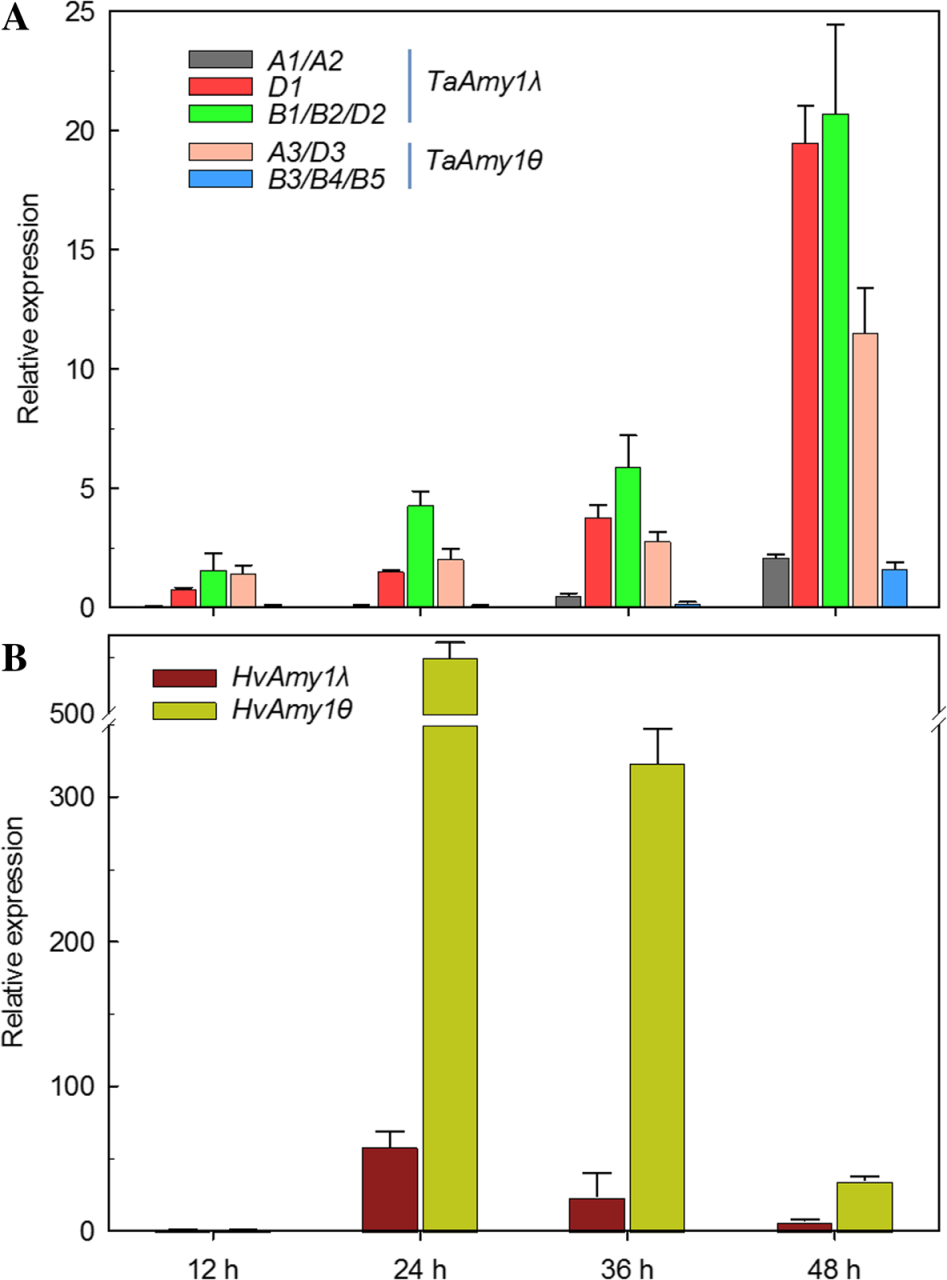
The relative expression levels of *Amy1* genes in wheat (cv. CS, **A**) and barley (cv. Morex, **B**) during germinating or early seedling stages. Three technical replicates were performed in each expression analysis. Error bars represent SE

We quantified transcript levels of *Amy1θ* and *Amy1λ* genes at germinating or early seedling stages. In bread wheat (cv. CS), the 11 full-length copies were divided into five subgroups, *TaAmy1-A1/A2*, *TaAmy1-D1*, *TaAmy1-B1/B2/D2*, *TaAmy1-A3/D3* and *TaAmy1-B3/B4/B5*, based on sequence homology. *TaAmy1-B1/B2/D2* transcripts were the most abundant, followed by *TaAmy1-D1* and *TaAmy1-A3/D3*, and subgroups *TaAmy1-A1/A2* and *TaAmy1-B3/B4/B5* were less expressed under all the four sampling points (Fig. 5A). In barley (cv. Morex), *HvAmy1θ* was significantly expressed at higher levels than *HvAmy1λ* (Fig. 5B).

**Fig. 6:**
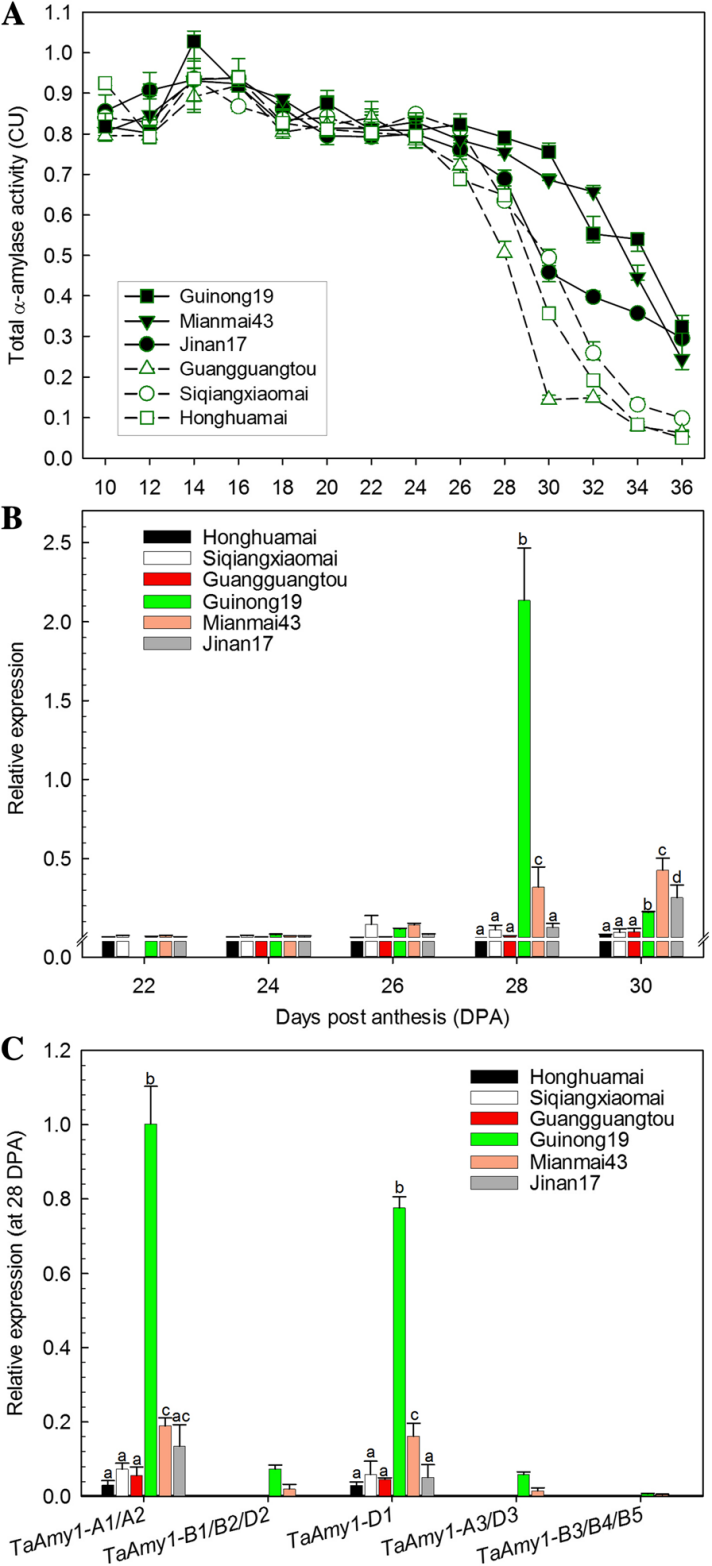
Dynamic changes of total α-amylase activities and expression levels of *TaAmy1* genes during grain development. (**a**) Changes of total α-amylase activities. (**b**) *TaAmy1* relative expression during late stages of grain development. (**c**) Relative expression of five *TaAmy1* subgroups at 28 DPA. Three technical replicates were performed in each expression analysis. Significance values were calculated using Duncan’s multiple range tests. Error bars represent SE

We introduced three wheat lines with high levels of α-amylase activities in ripe grains and three extreme low-level landraces for α-amylase assay (Additional file 7). In the grain development, all the six lines retained high α-amylase activities until 24 DPA; and from 26 DPA through to the end, high-level lines declined with lower rates and resulted in higher levels compared with the landraces (Fig. 6A). These changes prompted us to further investigate whether the *Amy1* copies were differentially expressed among these lines. Using an universal primer pair *TaAmy1-RT-*F/R, we observed an overall expression peak in Guinong19 at 28 DPA, which appeared slightly later in Mianmai43 and Jinan17. Their transcript levels were significantly higher than those of the other three landraces at 30 DPA (Fig. 6B). Further we performed copy-specific analysis at 28 DPA. It showed that the overall expression of *TaAmy1* in developing grains was largely contributed by *TaAmy1-A1/A2* and *TaAmy1-D1*, while *TaAmy1-B1/B2/D2*, *TaAmy1-A3/D3* and *TaAmy1-B3/B4/B5*) were hardly detected (Fig. 6C).

## Discussion

### Copy number variation of *Amy1* genes in bread wheat and barley

In this work, by molecular cloning and in silico analysis employing genomic resources generated recently, we isolated *Amy1* genes in bread wheat and its diploid progenitors, *T. urartu*, *Ae. speltoides* and *Ae. tauschii*, representing the A, B and D genomes, respectively (Table 1), although the real progenitor of B genome has been in debate for years [75, 76]. Compared with their corresponding counterparts of bread wheat, the *Amy1* genes of *T. urartu* and *Ae. tauschii* are highly conserved in terms of copy number and sequence identity, while those of *Ae. speltoides* are rather variable, which is consistent with previous reports [4]. Probably due to the amplification bias, we failed to experimentally isolate copies *TaAmy1-A3*, *TaAmy1-B4* and *TaAmy1-B5* (Table 1, Additional file 2) from CS, which could be retrieved from its whole genome sequence. However, we have confirmed their presence and the extremely high inter-cultivar conservation of each copy through sequencing more than 220 clones from 17 wheat lines.

In barley, eight *Amy1* copies were detected, which was consistent with the results recently presented by Mascher et al. [77]. However, some nomenclature confusion has arisen as genes for high-pI isoforms have been alternatively called *Amy1* [26] and *Amy2* [78] for many years. Radchuk et al. [23] submitted a barley α-amylase sequence named as *HvAMY3* (GenBank accession no. FN179391), which had an identity of 99% with *Amy6–4* and 95% with *Amy46*. Actually, it should not represent a new gene family [22], but belongs to the *Amy1* multigene family. Interestingly, in genomes of wheat and barley, we have found two interrupted *Amy1* fragments: *TaAmy1-B6* and *HvAmy1–6*, respectively. Analysis of their 4.1 kb upstream sequence indicates this truncating event might be caused by the insertion of a 1.2 kb Gypsy retrotransposon (Additional file 3B).

### Structural variation of *Amy1* loci in Pooideae

Syntenic analysis of *Amy1* loci revealed apparent structural variations between Pooideae and the other grass species analyzed in this study. Despite of the conserved linear gene order extensively reported in previous genome wide analyses [42, 79, 80], we found a segmental inversion around the *Amy1* loci occurring between 56.50 Mb - 59.11 Mb on chromosome 3 of *B. distachyon* comparing to corresponding region on chromosome 2 of rice (from 31.97 Mb to 33.40 Mb) (Fig. 3A). In corresponding regions on chromosomes 6 of barley and wheat, at least two segmental inversion events were observed (Fig. 3A, B). These structural alterations split the original *Amy1* locus into two separated loci: *Amy1θ* and *Amy1λ*, which resulted in significantly altered organization of *Amy1* comparing to those of some other grass species. The existence of these two *Amy1* loci was supported by the results of Nishikawa et al. [81] and Cheung et al. [82], who described two separated *TaAmy1* loci and five *TaAmy1* copies dispersed on chromosome 6B. Three individual Rye *Amy1* genes also provided some evidence of recombination and spanned a distance of 3 cM at the locus on chromosome 6RL [29]. The repeats invasion, especially LTR retrotransposon Copia (RLC) and Gypsy (RLG), and DNA transposon CACTA superfamily (DTC), also contributed to extension of intervals between *Amy1θ* and *Amy1λ* (Additional file 5).

Gene duplication is critical in supplying raw genetic materials to form gene families and producing new functions [83]. Copy number variation also reflects the dynamic genome evolutionary patterns. In wheat and barley, we have observed apparent evidence of tandem gene duplications, for example, four paralogous *TaAmy1θ* copies and two *TaAmy1λ* copies exist in chromosome 6B (Fig. 3A). These inter-group duplication events might occur prior to the intra-group duplications, as suggested by divergence time estimates (Table 2, Additional file 8). These results indicated that the Pooideae species underwent complex genome evolution.

**Fig. 7:**
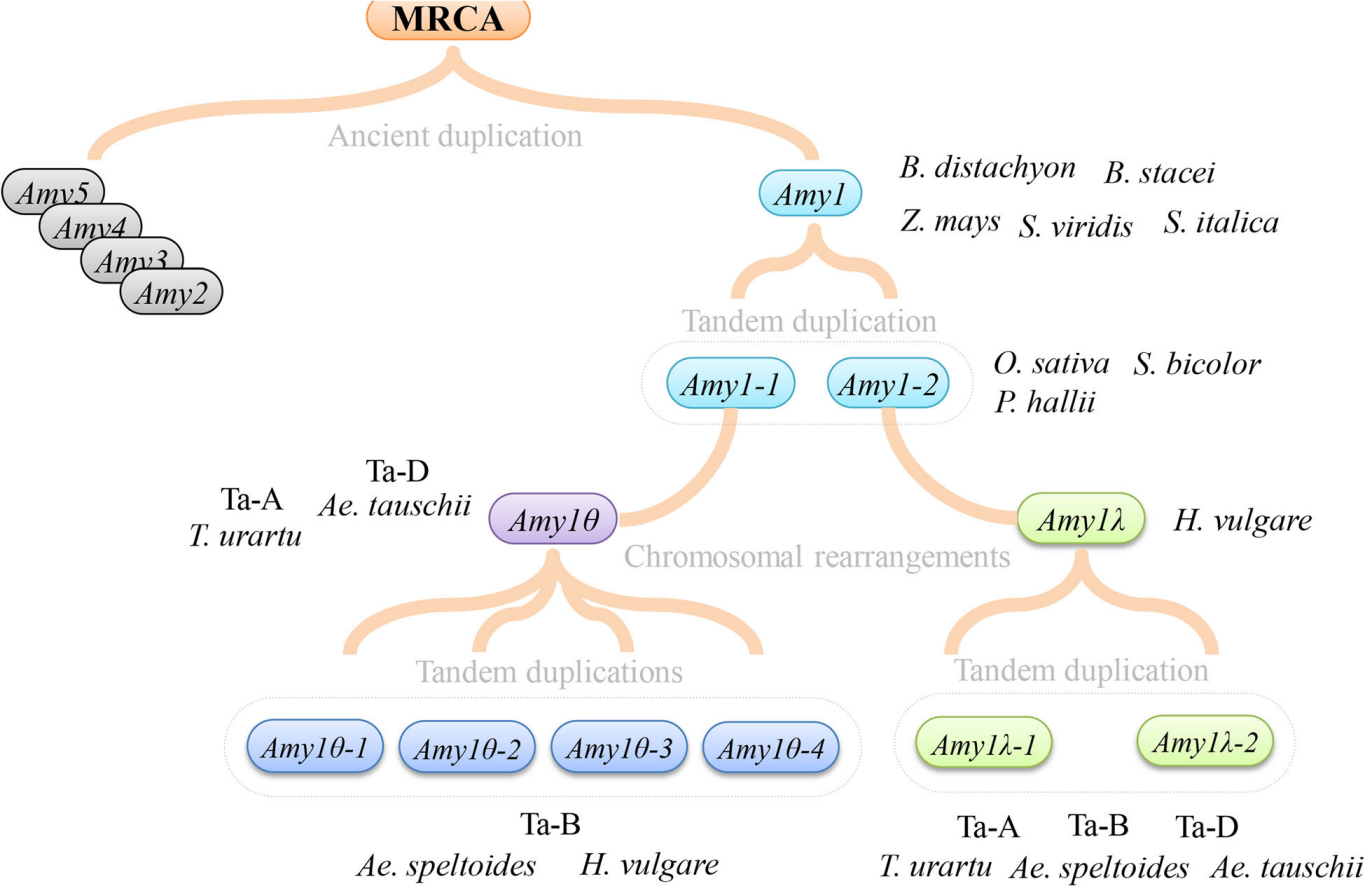
A proposed evolutionary model for *Amy1* locus from the most recent common ancestor (MRCA) among grass

Chromosomal distribution indicates that *Amy1* loci are located on the conserved block 5 (ancestral chromosome A4), which only experiences a whole genome duplication (WGD), followed by chromosomal breakage and shuffling before divergence of these three subfamilies (Pooideae, Ehrhartoideae and Panicoideae) in grass [41, 42, 79]. On the knowledge of grass establishment and *Amy1* structural variation, we proposed an evolutionary model of *Amy1* genes. As illustrated in Fig. 7, the original single-copy structural locus in grass (*Amy1*) retains in species of *B. distachyon*, *B. stacei*, *S. italica*, *S. viridis* and *Z. mays*, respectively. Two-copy species of *O. sativa*, *S. bicolor* and *P. hallii*, each experiences a tandem gene duplication event. In Triticeae species, *Amy1* is firstly duplicated into a pair of intermediate tandem repeats (*Amy1–1* and *Amy1–2*). Ever since this duplication, followed by chromosomal rearrangement, *Amy1–2* has evolved and diverged from *Amy1–1* under limited adaptive selections (Table 3). Two distinct loci, *Amy1θ* (*Amy1–1*) and *Amy1λ* (*Amy1–2*), have been eventually fixed in genomes. Subsequently, several rounds of other recent tandem duplications within each locus continue to enlarge this multigene family. Nevertheless, *Amy1* genes are absent in Dicot lineage, and should emerge after the branch of Dicots-Monocots, indicating the origin of the most recent common ancestor (MRCA) must have resulted from gene duplication.

### Divergence between *Amy1λ* and *Amy1θ* genes and functional importance

Not only the separation on physical positions, sequences of *Amy1θ* and *Amy1λ* genes are divergent. This is reflected by the phylogenetic analysis for that the *Amy1θ* and *Amy1λ* genes were clustered distinctly into two groups (Fig. 2), corresponding to groups G2 and G1, respectively. Additionally, length of the first intron varies between *Amy1θ* and *Amy1λ* genes of wheat and its progenitors. For example, the lengths of 370 bp or 380 bp were specifically appeared in *Amy1θ*, while this variation was not observed in *Amy1λ* genes (Additional file 9).

The fates (nonfunctionalization, neofunctionalization or subfunctionalization) of duplicated genes were strongly directed by adaptive selection [61, 84]. The nonsynonymous to synonymous substitution rate ratio (ω = dN / dS) is clearly recognized as a sensitive measure of positive selection at the nucleotide or protein levels. Using nine different codon-substitution models, we have identified two codon sites (Arg_97_ and Asn_233_) in *Amy1λ* genes under selection pressures (Table 3). The substitution of His97Arg was located on the 4th β-strand in domain B (Fig. 4), and did not share any functional evidence according to previous studies. The other codon site, Asp233Asn, was situated at the surface binding site (SBS1). SBS1 was involved in starch binding and substrate recognition [15, 20, 85]. This binding site owned two consecutive tryptophan residues (Additional file 6), which were characteristic of and functionally essential for cereal Amy1 or Amy2 isoforms [14, 15, 70]. Mutations on them strongly affected the ability of SBS1 binding to different starch types [85]. However, roles of these other surrounding residues (Asp233Asn, Gln226 and Val229 in Additional file 6) in starch binding or degradation have not yet been reported. Therefore, whether this substitution is functionally vital or not remains undiscovered. As the archetype *Amy1θ* genes commonly existing among grasses, the appearance of derivative *Amy1λ* genes indicates an unique gene duplication event in Triticeae species, and possibly implicated the potential subfunctionalization of *Amy1* after divergence of the two groups.

Patterns of gene expression are usually associated with functional differentiation. In the present work, all the five subgroups of *TaAmy1* and two subgroups of *HvAmy1* are active in transcription during seed germination (Fig. 5A, B), indicating that the seed germination is triggered by the coordinated expressions of multiple *Amy1θ* and *Amy1λ* genes. In barley, *HvAmy1θ* (at least four copies) exhibited significantly higher abundance than that of *HvAmy1λ* (one copy) during germination (Fig. 5B). Considering great difference on copy number, the differential expression levels most likely owe to dosage effect. In wheat, both during germination and in the developmental stages, our study indicated that *TaAmy1λ* and *TaAmy1θ* genes were differentially expressed, and *TaAmy1λ* copies seemed to largely contribute to the total abundance of *TaAmy1* genes. For example, *TaAmy1-D1* and *TaAmy1-B1/B2/D2* during germination, and *TaAmy1-A1/A2* and *TaAmy1-D1* in the developing grains were the main *TaAmy1λ* subgroups abundantly expressed (Fig. 5A and Fig. 6B, C).

As changes in regulatory sequences affect transcript levels and result in expression divergence in duplicated genes [86], we then compared the 18 promoter sequences (approximately 200 bp – 250 bp upstream of the ‘tata’ box) from bread wheat and barley. Previous reports indicated both *Amy1* and *Amy2* genes owned three GA-responsive elements [24, 31, 87]. Both the pyrimidine box and the ‘taacaaac’ box were conserved between *Amy1θ* and *Amy1λ* genes, while the ‘tatccac/t’ box showed a nucleotide substitution of C (*Amy1θ*) to T (*Amy1λ*) (Additional file 10). This substitution was also found in a highly conserved element (‘tatccatgcagtg’ box) of *Amy32b*, a representative of low-pI *Amy2* gene family [87]. We also sequenced promoter (1.1 kb) and coding sequences of *TaAmy1-A1*, *TaAmy1-A2* and *TaAmy1-D1* from these six investigated wheat lines (Additional file 11), and failed to detect any informative variation associated with this apparent expression divergence. Thus, the regulation mechanisms of the elevated expression levels or activities, as suggested by Farrell et al. [88] and Yang et al. [39], needs to be further uncovered.

## Conclusion

In this study, we present comprehensive analyses of *Amy1* genes in wheat and barley. Copy number extension of *Amy1* genes is evident. Under actions of tandem gene duplication and chromosome rearrangement, the original *Amy1* locus was divided into two spatially structural loci (*Amy1θ* and *Amy1λ*). Potential functional divergence between them is clear according to their sequence mutations and expression differentiations. Genetically, the *Amy1* multigene family originates from a single-copy structural locus, and its expansion pattern provides a divergent model during gene duplication and evolution. For agronomical practices, the observed expression differentiation of duplicated copies, and functional divergence between *Amy1θ* and *Amy1λ* genes will help to better understand the mechanism underlying the dynamic changes of α-amylase activity during germination or seed maturation, and provide clues or orientations for dissection of genetic factors impacting α-amylase activities in wheat and barley, which will be helpful to further identification of alleles favored for better yield and processing qualities.

## Additional files

Additional file 1: **Table S1.** Primers (F, forward; R, reverse) used in this study. (DOCX 17 kb)

Additional file 2: **Figure S1.** Neighbor-Joining clustering analysis of 125 isolated sequences and 11 full-length copies retrieved from IWGSC WGA v0.4, consisting of 55 isolates from CS (14 *TaAmy1-A1*; 3 *TaAmy1-A2*; 3 *TaAmy1-B1*; 10 *TaAmy1-B2*; 7 *TaAmy1-B3*; 9 *TaAmy1-D1*; 7 *TaAmy1-D2*; 2 *TaAmy1-D3*), 19 from PI428191 (9 *TuAmy1-A1*; 9 *TuAmy1-A2*; 1 *TuAmy1-A3*), 18 isolates from PI542268 (2 *AesAmy1-B1*; 3 *AesAmy1-B2*; 6 *AesAmy1-B3*; 2 *AesAmy1-B4*; 4 *AesAmy1-B5*; 1 *AesAmy1-B6*) and 33 isolates from AS2404 (29 *AetAmy1-D1*; 1 *AetAmy1-D2*; 3 *AetAmy1-D3*). Copies *TaAmy1-A3*, *TaAmy1-B4* and *TaAmy1-B5* were failed to isolate, because of lack of enough clones and/or the existence of amplification bias. (TIF 1647 kb)

Additional file 3: **Figure S2.** Existence and structure of the truncated copy *TaAmy1-B6*. (**A**) Amplification products of *TaAmy1-B6* (M marker; 1 CS; 2 PI428191; 3 PI542268; 4 AS2404; 5 N6AT6B; 6 N6AT6D; 7 N6BT6A; 8 N6BT6D; 9 N6DT6A; 10 N6DT6B). (**B**) The truncated structure of *TaAmy1-B6*. (TIF 31 kb)

Additional file 4: **Table S2.** Estimation of *Amy1* genetic distances in grass. (DOCX 16 kb)

Additional file 5: **Figure S3.** Repeat annotation of intervals between *TaAmy1θ* and *TaAmy1λ*. The identification of repetitive elements was analyzed by using a local BLASTN search against the non-redundant dataset of Triticeae Repeats (http://botserv2.uzh.ch/kelldata/trep-db/index.html). LTR retrotransposons: Copia (RLC), Gypsy (RLG) and unclassified LTR (RLX); non-LTR retrotransposons: SINE (SIX) and LINE (RIX). DNA transposons: CACTA superfamily (DTC), Mutator superfamily (DTM), PIF/Harbinger superfamily (DTH), Tc1/Mariner superfamily (DTT), hAT superfamily (DTA), MITEs (DXX), Helitron (DHH) and unclassified (DTX), and unclassified elements (XXX). (TIF 648 kb)

Additional file 6: **Figure S4.** The substitution of Asp233Asn in the SBS1 region. Overall structure of Amy1 was presented in complex with substrate analogues: DAF-BGC and AF1 ligands. Dashed line boxes (from up to down) represented the two starch binding sites: the main active site and the surface binding site (SBS1), respectively. DAF: 4,6-dideoxy-4-{[(1S,5R,6S)-3-formyl-5,6-dihydroxy-4-oxocyclohex-2-en-1-yl]amino}-α-D-xylo-hex-5-enopyranose, BGC: β-D-glucose, AF1: 4,6-dideoxy-4-{[(1S,4S,5S,6S)-4,5,6-trihydroxy-3-(hydroxymethyl)cyclohex-2-en-1-yl]amino}-β-D-glucopyranose. Calcium ions were represented as green balls. Helices were colored in dark cyan, strands in gold, coils in gray and ligands & C in white. Red arrows represented the two tryptophan residues in SBS1, and the black indicated the Asp233Asn substitution. (TIF 3122 kb)

Additional file 7: **Figure S5.** Residual α-amylase activities of ripe wheat grains under three natural conditions. (TIF 550 kb)

Additional file 8: **Table S3.** Estimation of duplication and divergence time within groups. (DOCX 17 kb)

Additional file 9: **Figure S6.** Length variation of the first intron of 28 *Amy1* genes. (TIF 949 kb)

Additional file 10: **Figure S7.** Sequence alignment of 18 *Amy1* genes in the promoter region, approximately 200–250 bp from the ‘tata’ box. (TIF 399 kb)

Additional file 11: Promoter and coding sequences of *TaAmy1-A1*, *TaAmy1-A2* and *TaAmy1-D1* isolated from six wheat lines. (TXT 51 kb)

## Abbreviations

BEB: Bayes empirical bayes
CS: Chinese spring
CTAB: cetyl trimethylammonium bromide
DPA: days post anthesis
GA: gibberellic acid
GARE: gibberellic acid response elements
IEF: isoelectric focusing
LMA: late maturity α-amylase
LRT: Likelihood ratio test
MYA: million years ago
NEB: Naive empirical bayes
NT: nullisomic-tetrasomic
PAGE: polyacrylamide gel electrophoresis
PHS: pre-harvest sprouting
pI: isoelectric point
RFLP: restriction fragment length polymorphism
SBS1: starch granule binding surface site
WGD: whole genome duplication
