# Supplementary material for: Structural organization and functional divergence of high isoelectric point α-amylase genes in bread wheat (Triticum aestivum L.) and barley (Hordeum vulgare L.)
Source: BMC Genet. 2019 Mar 7;20:25. doi: 10.1186/s12863-019-0732-1 (PMC6404323; doi:10.1186/s12863-019-0732-1)
Supplement: Supplementary file 1 — Table S1. Primers (F, forward; R, reverse) used in this study. (DOCX 17 kb) [file 12863_2019_732_MOESM1_ESM.docx]

**Table S1** Primers (F, forward; R, reverse) used in this study

| Name | Sequence (5' - 3') | Use of primer |
| --- | --- | --- |
| *TaAmy1*-F | TGAAT(G/C)CATCAGTTCTCCA(C/T)C | gene cloning |
| *TaAmy1*-R | G(C/T)CCCTCATCCTCACTTTTACATGG |  |
| *TaAmy1-B6*-F | GTTCACCCACCATATGCTATC | gene cloning |
| *TaAmy1-B6*-R | GTCCATACCTCAGCCTTTC |  |
| *TaAmy1-A1/A2*-F | ACGCACCAGCTGAATCCATC | gene cloning |
| *TaAmy1-A1/A2*-R | GGTGTG(A/G)CATTCATATATAGG |  |
| *TaAmy1-D1*-F | GCAGCACACTATAAATACCTG | gene cloning |
| *TaAmy1-D1*-R | CTACGTGGACATCATGAGCTC |  |
| *TaAmy1-A1*-pF | GTAATCTGCCACACGGTTG | promoter cloning |
| *TaAmy1-A1*-pR | ACGAGTGTTTGTTCGCCATG |  |
| *TaAmy1-A2*-pF | GTCGAAGGACATTGCCTT | promoter cloning |
| *TaAmy1-A2*-pR | ACGAGTGTTTGTTCGCCATG |  |
| *TaAmy1-D1*-pF | TGAGCTACGGTGCAATCTTG | promoter cloning |
| *TaAmy1-D2*-pR | ACGAGTGTTTGTTCGCCATG |  |
| *TaAmy1-*RT-F | GGATACACAGTGA(A/G)AGCAAG | gene expression |
| *TaAmy1*-RT-R | GCGTC(A/G)GCCTCTATGATTTG |  |
| *TaAmy1-A1/A2*-RT-F | CAAGGCTTCAAGGTGGT | gene expression |
| *TaAmy1-A1/A2*-RT-R | GGTGT(A/G)ACATTCATATATAGG |  |
| *TaAmy1-B1/B2/D2*-RT-F | CGCACACGGCAAAGACTAT | gene expression |
| *TaAmy1-B1/B2/D2*-RT-R | GTGGACAACATGACTAATTTGC |  |
| *TaAmy1-D1*-RT-F | ATACGATGTCGGGCACCTCAT | gene expression |
| *TaAmy1-D1*-RT-R | GTGGACATCATGAGCTCCGGTAA |  |
| *TaAmy1-A3/D*3-RT-F | CAAAATTACCAAAGCAGCTCTACGAG | gene expression |
| *TaAmy1-A3/D3*-RT-R | ACTTTTACATGGAGGAAGTACTAAATCGTAC |  |
| *TaAmy1-B3/B4/B5*-RT-F | GATGTGGGACACCTCATT | gene expression |
| *TaAmy1-B3/B4/B5*-RT-R | CCAATATGTATCACGTACACATAG |  |
| *HvAmy1*-G1/G2-RT-F | GAGATCGACGGCAAGGTCAT | gene expression |
| *HvAmy1*-G1-RT-R | AGCTCGGACTAATTTGTAGAG |  |
| *HvAmy1*-G2-RT-R | CATATAGGAACTTGTAGAGCTGC |  |
