# Supplementary figures and images for: Structural organization and functional divergence of high isoelectric point α-amylase genes in bread wheat (Triticum aestivum L.) and barley (Hordeum vulgare L.)
Source: BMC Genet. 2019 Mar 7;20:25. doi: 10.1186/s12863-019-0732-1 (PMC6404323; doi:10.1186/s12863-019-0732-1)

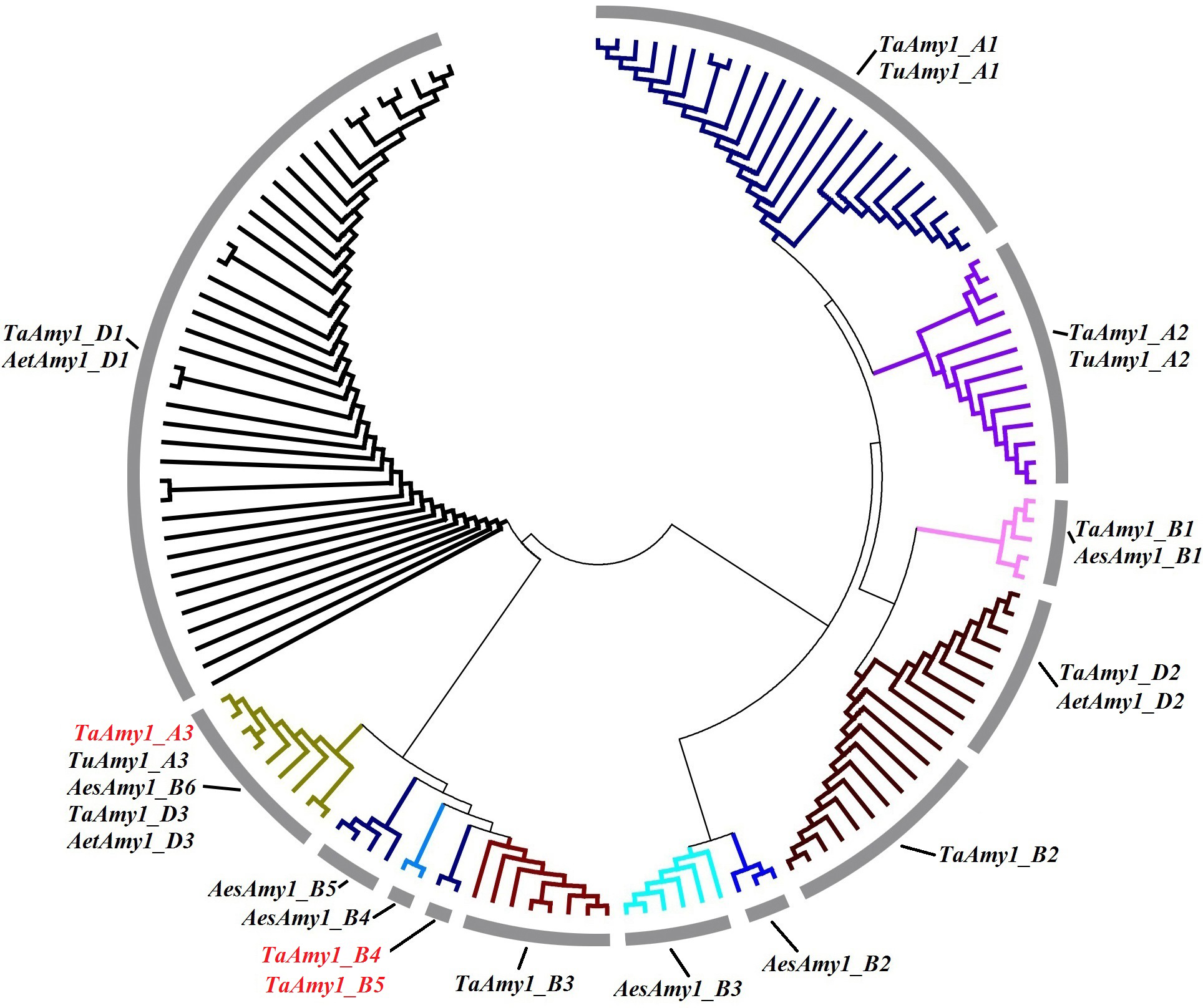

Supplement: Supplementary file 2 — Figure S1. Neighbor-Joining clustering analysis of 125 isolated sequences and 11 full-length copies retrieved from IWGSC WGA v0.4, consisting of 55 isolates from CS (14 TaAmy1-A1; 3 TaAmy1-A2; 3 TaAmy1-B1; 10 TaAmy1-B2; 7 TaAmy1-B3; 9 TaAmy1-D1; 7 TaAmy1-D2; 2 TaAmy1-D3), 19 from PI428191 (9 TuAmy1-A1; 9 TuAmy1-A2; 1 TuAmy1-A3), 18 isolates from PI542268 (2 AesAmy1-B1; 3 AesAmy1-B2; 6 AesAmy1-B3; 2 AesAmy1-B4; 4 AesAmy1-B5; 1 AesAmy1-B6) and 33 isolates from AS2404 (29 AetAmy1-D1; 1 AetAmy1-D2; 3 AetAmy1-D3). Copies TaAmy1-A3, TaAmy1-B4 and TaAmy1-B5 were failed to isolate, because of lack of enough clones and/or the existence of amplification bias. (TIF 1647 kb) [file 12863_2019_732_MOESM2_ESM.tif]

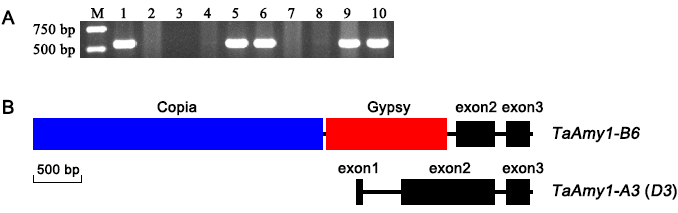

Supplement: Supplementary file 3 — Figure S2. Existence and structure of the truncated copy TaAmy1-B6. (A) Amplification products of TaAmy1-B6 (M marker; 1 CS; 2 PI428191; 3 PI542268; 4 AS2404; 5 N6AT6B; 6 N6AT6D; 7 N6BT6A; 8 N6BT6D; 9 N6DT6A; 10 N6DT6B). (B) The truncated structure of TaAmy1-B6. (TIF 31 kb) [file 12863_2019_732_MOESM3_ESM.tif]

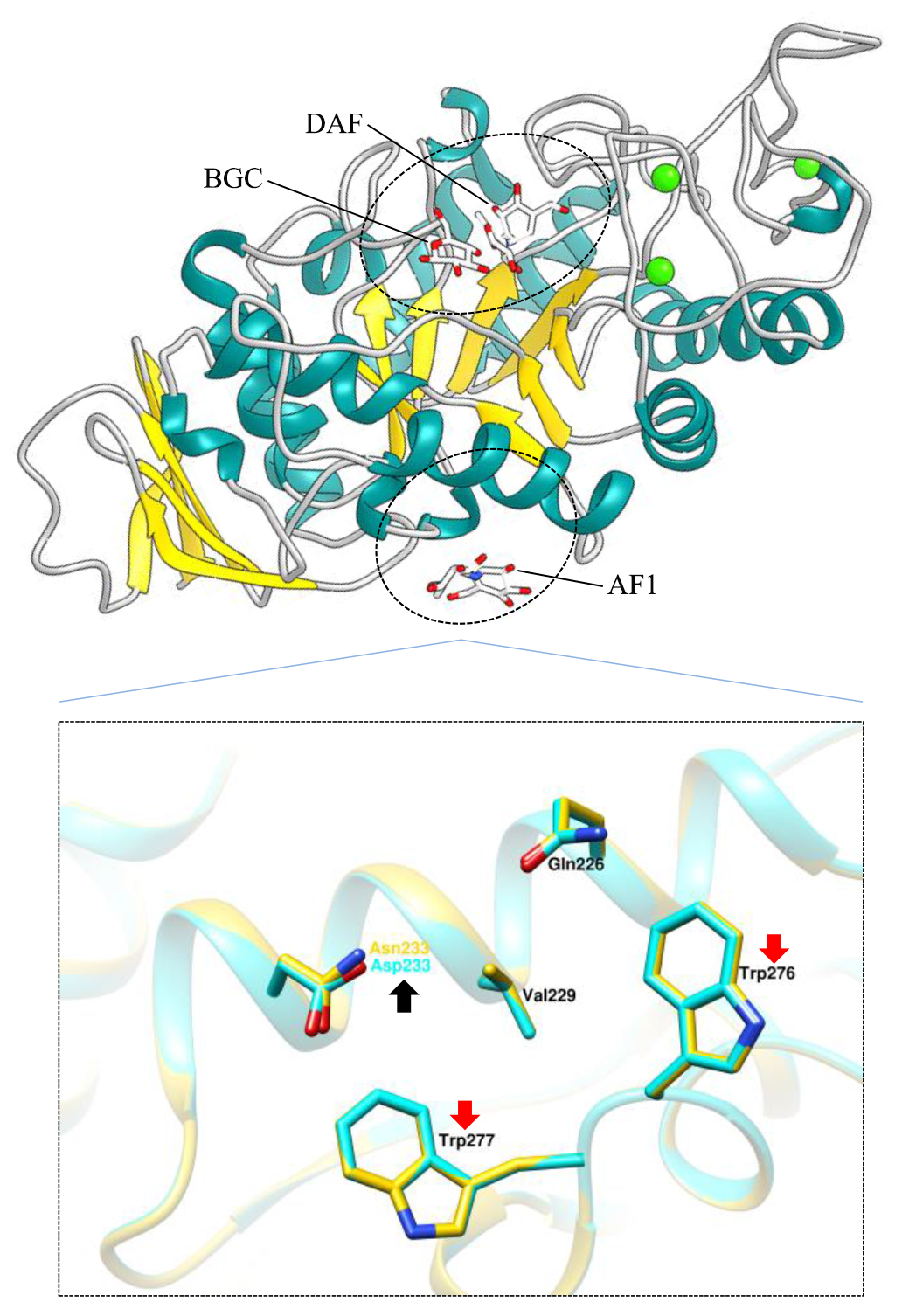

Supplement: Supplementary file 6 — Figure S4. The substitution of Asp233Asn in the SBS1 region. Overall structure of Amy1 was presented in complex with substrate analogues: DAF-BGC and AF1 ligands. Dashed line boxes (from up to down) represented the two starch binding sites: the main active site and the surface binding site (SBS1), respectively. DAF: 4,6-dideoxy-4-{[(1S,5R,6S)-3-formyl-5,6-dihydroxy-4-oxocyclohex-2-en-1-yl]amino}-α-D-xylo-hex-5-enopyranose, BGC: β-D-glucose, AF1: 4,6-dideoxy-4-{[(1S,4S,5S,6S)-4,5,6-trihydroxy-3-(hydroxymethyl)cyclohex-2-en-1-yl]amino}-β-D-glucopyranose. Calcium ions were represented as green balls. Helices were colored in dark cyan, strands in gold, coils in gray and ligands & C in white. Red arrows represented the two tryptophan residues in SBS1, and the black indicated the Asp233Asn substitution. (TIF 3122 kb) [file 12863_2019_732_MOESM6_ESM.tif]

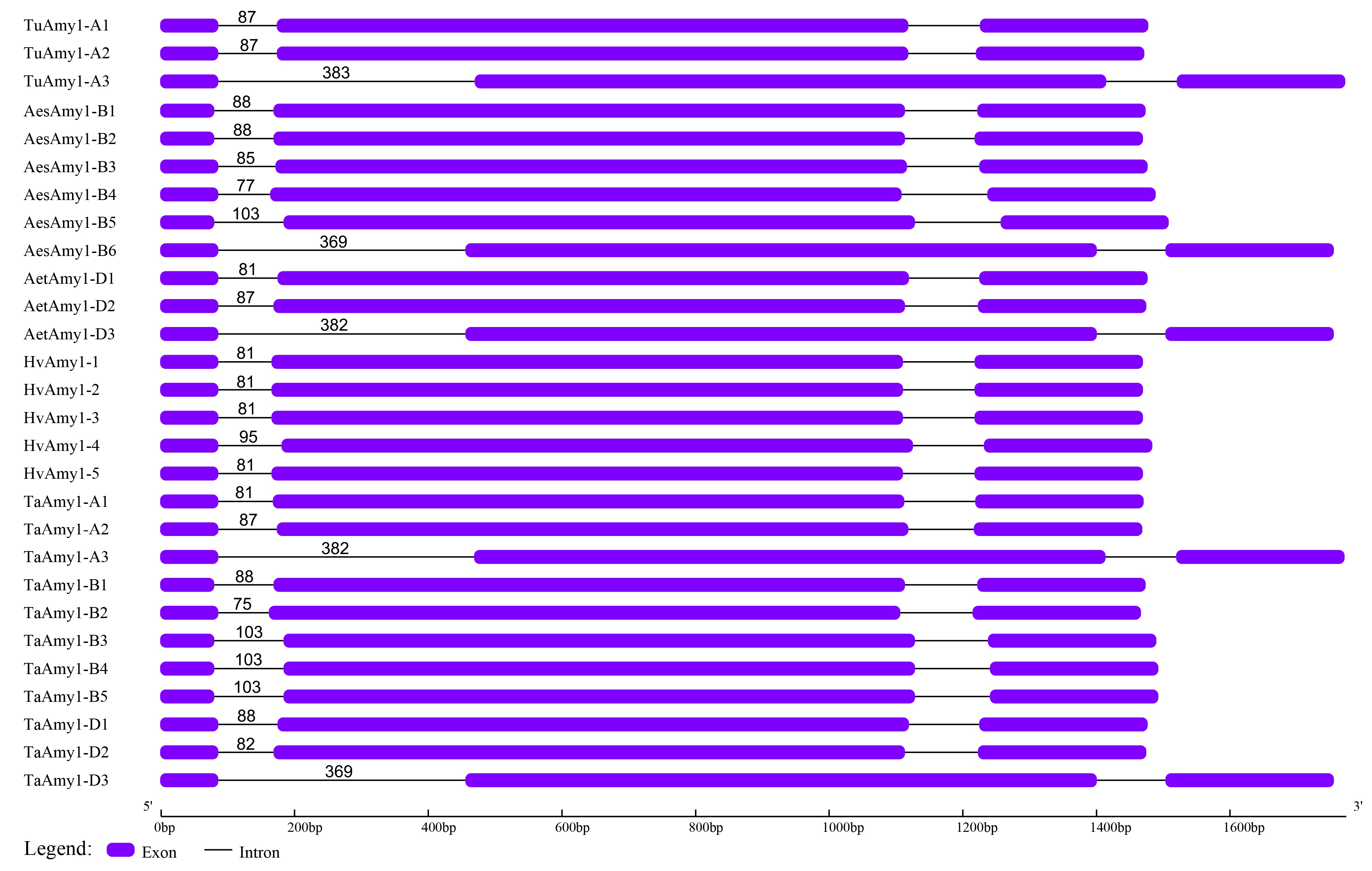

Supplement: Supplementary file 9 — Figure S6. Length variation of the first intron of 28 Amy1 genes. (TIF 949 kb) [file 12863_2019_732_MOESM9_ESM.tif]

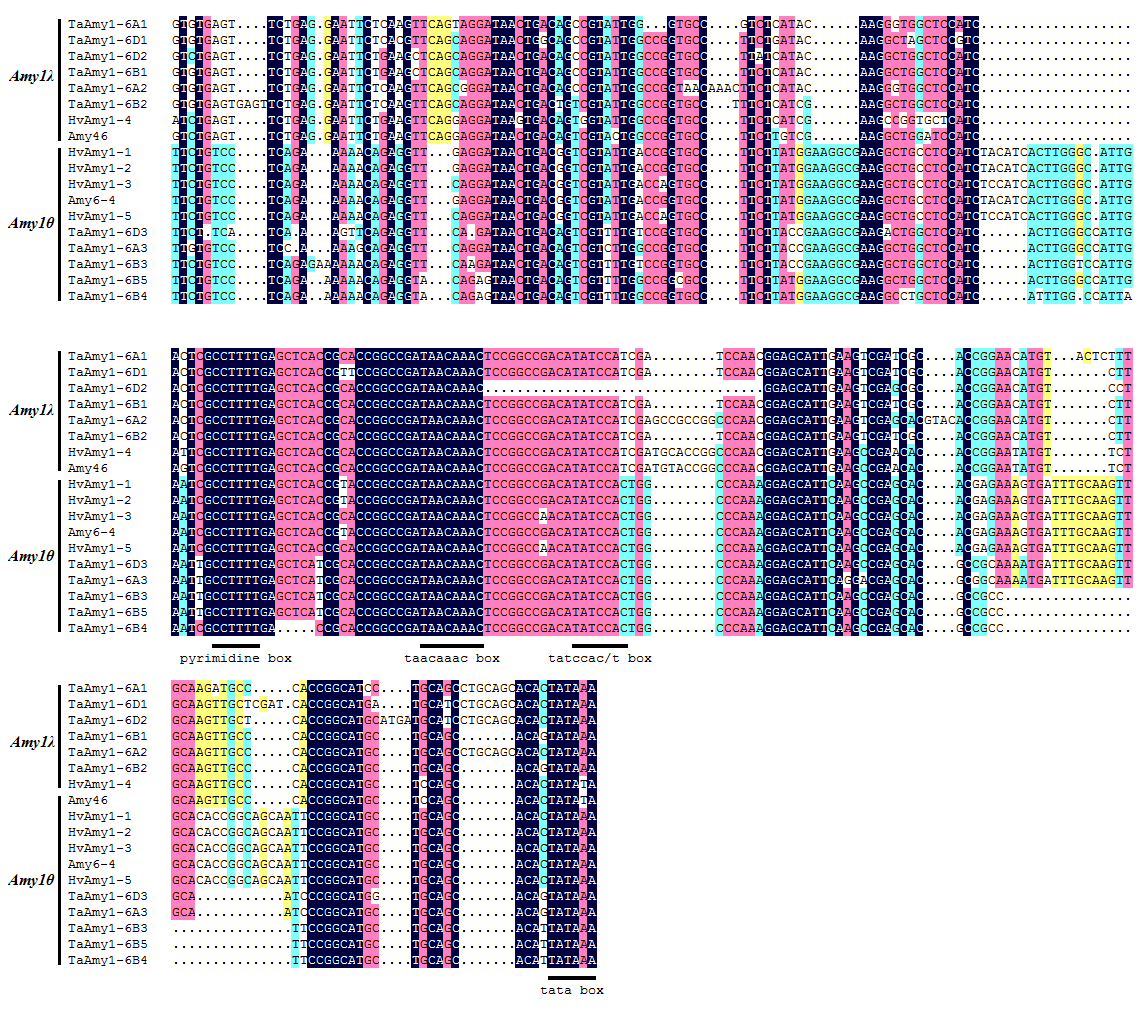

Supplement: Supplementary file 10 — Figure S7. Sequence alignment of 18 Amy1 genes in the promoter region, approximately 200–250 bp from the ‘tata’ box. (TIF 399 kb) [file 12863_2019_732_MOESM10_ESM.tif]
