# Supplementary material for: Structural organization and functional divergence of high isoelectric point α-amylase genes in bread wheat (Triticum aestivum L.) and barley (Hordeum vulgare L.)
Source: BMC Genet. 2019 Mar 7;20:25. doi: 10.1186/s12863-019-0732-1 (PMC6404323; doi:10.1186/s12863-019-0732-1)
Supplement: Supplementary file 4 — Table S2. Estimation of Amy1 genetic distances in grass. (DOCX 16 kb) [file 12863_2019_732_MOESM4_ESM.docx]

**Table S2** Estimation of genetic distances in grass

|  | in group | G3**^a^** | G1 | G2 | G4**^b^** | G5**^c^** |
| --- | --- | --- | --- | --- | --- | --- |
| G3**^a^** | 0.05 |  | 0.20 | 0.19 | 0.21 | 0.23 |
| G1 | 0.03 |  |  | 0.05 | 0.20 | 0.23 |
| G2 | 0.02 |  |  |  | 0.18 | 0.20 |
| G4**^b^** | 0.06 |  |  |  |  | 0.18 |
| G5**^c^** | 0.10 |  |  |  |  |  |

**^a^** The Pooideae-*Brachypodium* clade. **^b^** The Ehrhartoideae clade. **^c^** The Panicoideae clade.
