# Supplementary material for: Structural organization and functional divergence of high isoelectric point α-amylase genes in bread wheat (Triticum aestivum L.) and barley (Hordeum vulgare L.)
Source: BMC Genet. 2019 Mar 7;20:25. doi: 10.1186/s12863-019-0732-1 (PMC6404323; doi:10.1186/s12863-019-0732-1)
Supplement: Supplementary file 8 — Table S3. Estimation of duplication and divergence time within groups. (DOCX 17 kb) [file 12863_2019_732_MOESM8_ESM.docx]

**Table S3** Estimation of duplication and divergence time within groups

| Pairwise comparison | dS | T (MYA) |
| --- | --- | --- |
| *TaAmy1-A2* vs *TaAmy1-A1* | 0.2602 ± 0.0515 | 20.0 ± 4.0 |
| *TuAmy1-A2* vs *TuAmy1-A1* | 0.2062 ± 0.0449 | 15.9 ± 3.5 |
| *TaAmy1-B2* vs *TaAmy1-B1* | 0.2587 ± 0.0526 | 19.9 ± 4.0 |
| *AesAmy1-B2* vs *AesAmy1-B1* | 0.1807 ± 0.0401 | 13.9 ± 3.1 |
| *AesAmy1-B3* vs *AesAmy1-B1* | 0.2042 ± 0.0442 | 15.7 ± 3.4 |
| *AesAmy1-B3* vs *AesAmy1-B2* | 0.1739 ± 0.0411 | 13.4 ± 3.2 |
| *TaAmy1-D2* vs *TaAmy1-D1* | 0.4542 ± 0.0792 | 34.9 ± 6.1 |
| *AetAmy1-D2* vs *AetAmy1-D1* | 0.4542 ± 0.0792 | 34.9 ± 6.1 |
| *TaAmy1-B4* vs *TaAmy1-B3* | 0.0139 ± 0.0099 | 1.1 ± 0.8 |
| *TaAmy1-B5* vs *TaAmy1-B4* | 0.2392 ± 0.0491 | 18.4 ± 3.8 |
| *TaAmy1-B5* vs *TaAmy1-B3* | 0.2200 ± 0.0468 | 16.9 ± 3.6 |
| *AesAmy1-B5* vs *AesAmy1-B4* | 0.0662 ± 0.0229 | 5.1 ± 1.8 |
| *AesAmy1-B6* vs *AesAmy1-B5* | 0.2664 ± 0.0522 | 20.5 ± 4.0 |
| *AesAmy1-B6* vs *AesAmy1-B4* | 0.2394 ± 0.0486 | 18.4 ± 3.7 |
| *HvAmy1-2* vs *HvAmy1-1* | 0 | 0 |
| *HvAmy1-3* vs *HvAmy1-2* | 0 | 0 |
| *HvAmy1-3* vs *HvAmy1-1* | 0 | 0 |
| *HvAmy1-5* vs *HvAmy1-3* | 0 | 0 |
| *HvAmy1-5* vs *HvAmy1-2* | 0 | 0 |
| *HvAmy1-5* vs *HvAmy1-1* | 0 | 0 |
